# Maternal outcome in HELLP syndrome requiring intensive care management in a Turkish hospital

**DOI:** 10.1590/S1516-31802006000200007

**Published:** 2006-03-02

**Authors:** Mehmet Armagan Osmanagaoglu, Selen Osmanagaoglu, Hülya Ulusoy, Hasan Bozkaya

**Keywords:** Maternal mortality, Pre-eclamp-sia, HELLP syndrome, Intensive care, High-risk pregnancy, Mortalidade materna, Pré-eclâmpsia, Síndrome HELLP, Cuidados intensivos, Gravidez de alto risco

## Abstract

**CONTEXT AND OBJECTIVE:**

Despite the development of tertiary care facilities, intensive care and advanced blood banking techniques, pregnancy-related hypertensive disorders are the main cause of maternal mortality in most countries. Our purpose was to determine maternal outcome in pregnancies complicated by HELLP syndrome (hemolysis, elevated liver enzymes and low platelet count) that required intensive care management.

**DESIGN AND SETTING:**

Retrospective study at Department of Obstetrics and Gynecology, and Department of Anesthesiology and Reanimation, Karadeniz Technical University, Trabzon, Turkey.

**METHODS:**

37 patients with HELLP syndrome admitted to the obstetric intensive care unit were analyzed retrospectively from 1992 to 2004.

**RESULTS:**

All patients were hypertensive, with mean Glasgow coma score (GCS) of 11 ± 3.96. Mean gestational age at delivery was 32 ± 4.09 weeks. Delivery was vaginally in nine and by cesarean section in 27 patients. General anesthesia was used in 12 and spinal anesthesia in 25 patients. Maternal morbidity included acute renal failure (11%), disseminated intravascular coagulation (5%), acute lung edema (3%), severe ascites (11%), pleural effusion (3%), adult respiratory distress syndrome (11%), abruptio placenta (11%), cerebral edema (8%) and cerebral hemorrhage (40%). All patients required transfusions using blood products. There were 11 maternal deaths (30%).

**CONCLUSION:**

Because of high maternal mortality and morbidity found among patients with HELLP syndrome, standard antenatal follow-up protocols should be applied, so as to obtain early diagnosis and improve the speed of transfer to obstetric departments with expertise in this field.

## RESUMO

**CONTEXTO E OBJETIVO:**

Apesar do desenvolvimento de instalações terciárias de cuidado e tratamento intensivo e de técnicas avançadas de transfusão de sangue, desordens hipertensivas da gravidez são a causa principal de mortalidade materna na maioria dos países. Nosso objetivo foi determinar o resultado materno nas gravidezes complicadas pela síndrome HELLP (hemólise, enzimas hepáticas elevadas e contagem de plaquetas baixa) que exigiram cuidados intensivos.

**TIPO DE ESTUDO E LOCAL:**

Estudo retrospectivo realizado na Karadeniz Technical University, Department of Obstetrics and Gynecology, and Department of Anaesthesiology and Reanimation, Trabzon, Turquia.

**MÉTODOS:**

37 pacientes com a síndrome HELLP admitidas à unidade de cuidado intensivo obstétrico foram analisadas retrospectivamente entre 1992 e 2004.

**RESULTADOS:**

Todasas pacientes eram hipertensas, com escala Glasgow de coma média de 11 ± 3,96. A idade gestacional média no parto foi de 32 ± 4,09 semanas. O parto foi vaginal em 9 e por cesárea em 27 pacientes. Anestesia geral foi usada em 12 e loco-regional em 25 pacientes. Morbidades maternas incluíram a falência renal aguda (11%), coagulação intravascular disseminada (5%), edema agudo do pulmão (3%), ascite grave (11%), o derrame pleural (3%), síndrome respiratória aguda grave (11%), descolamento prematuro de placenta (11%), edema cerebral (8%) e hemorragia cerebral (40%). Todas as pacientes necessitaram de transfusão de produtos do sangue. Ocorreram 11 (30%) mortes maternas.

**CONCLUSÕES:**

Devido à alta morbidade e mortalidade maternas encontradas nas pacientes com síndrome HELLP, protocolos antenatais de acompanhamento devem ser aplicados de modo a se obter diagnóstico precoce e a de apressar a transferência para um departamento obstétrico onde a equipe de profissionais tenha perícia no campo.

## INTRODUCTION

Maternal mortality, presented as a ratio, measures obstetric risk per 100,000 live births and has been defined internationally as the total number of deaths of women during pregnancy or within 42 days after the end of pregnancy.^1^ Pregnancy-related hypertensive disorders are the main cause of maternal mortality in most countries. Data from developed countries show maternal mortality of about 0.1% due to preeclampsia, in which the majority of cases were complicated by the HELLP syndrome (hemolysis, elevated liver enzymes and low platelet count).^1^ Despite the development of tertiary care facilities, intensive care and advanced blood banking techniques, maternal and neonatal deaths continue to occur in association with HELLP syndrome.^2-4^ The reported maternal mortality due to HELLP syndrome ranges from 0% to 24%.^4^

## OBJECTIVE

In this present study our aim was to determine maternal morbidity and mortality among women with HELLP syndrome who required transfer for critical care.

## METHODS

This was a retrospective study carried out within the Department of Obstetrics and Gynecology and Department of Anesthesiology and Reanimation, School of Medicine, Karadeniz Technical University, Trabzon, Turkey.

All patients with HELLP syndrome (n = 37) admitted to the Department of Obstetrics and Gynecology between January 1992 and June 2004 were analyzed retrospectively. Shortly after delivery, all the patients were cared for in the intensive care unit (ICU).

The clinical examination included temperature measurement, non-invasive pressure monitoring, heart rate and urinary output, and routine laboratory evaluations included serial measurement of liver function tests, complete blood cell count, coagulation profile, and renal function tests. Neurological examination included the utilization of the Glasgow coma score (GCS), a widely used scoring system for quantifying the level of consciousness following traumatic brain injury. It evaluates the best eye opening response, verbal response and motor response.

Computerized tomography or magnetic resonance imaging was performed as indicated by focal neurological signs such as recurrent seizures after initiation of anticonvulsant therapy, coma and unusual behavioral changes. Abdominal ultrasonography was performed to investigate possible presence of subcapsular liver hematoma or rupture, if indicated. Acute renal failure was diagnosed in the presence of oliguria or four hours of anuria in association with severely reduced renal function: raised serum creatine (≥ 1.13 mg/dl) and serum urea levels, and diminished creatinine clearance (≤ 20 ml/min). Diagnoses of acute lung edema or pleural effusion were utilized as determinants of clinical status or radiological investigation of the cases. Disseminated intravascular co-agulation (DIC) was defined as the presence of low platelets (< 100 × 10^9^/l), low fibrinogen (< 300 mg/dl), prolonged prothrombin time (≥ 14 sec), partial thromboplastin time ≥ 40 sec, and estimates of fibrinogen degradation products or D-dimer ≥ 800 ng/ml.^5^ Gestational age was determined from the last menstrual period, uterus size upon admission, or early sonography, if obtained.

The diagnosis of HELLP syndrome was made using the criteria established by Sibai,^4^ i.e. on the basis of hemolysis, abnormal peripheral blood smear, increased lactic dehydrogenase (LDH) (> 600 U/I), increased total bilirubin (> 1.2 mg/dl), elevated liver enzymes [increased plasma aspartate amino transferase (AST) > 70 U/I], low platelets (platelet count < 100 × 10^9^/l).^2^ To prevent and control seizures, women with HELLP syndrome received magnesium sulfate as a 6 g intravenous loading dose followed by a 1 g intravenous dose per hour. α-methyldopa, labetalol, nifedipine and sodium nitroprusside, or a combination of these drugs, were administered to control severe hypertension and to maintain diastolic blood pressure between 90 and 100 mmHg. Blood and blood products were used to correct coagulation abnormalities as needed, and when hemoglobin concentration was < 8 g/dl. Oliguria was initially managed using colloid plasma substitute fluid challenges. If there was no response, infusion of dopamine 200 mg, 2 µg/kg/h, was prescribed and, if necessary, a central venous catheter was inserted to aid in hemodynamic management. For women who were transferred to the renal unit for dialysis, renal clinical charts were analyzed to find whether renal function returned to normal levels.

## RESULTS

During the study period, the total number of deliveries in our Hospital was 4,485 and there were 542 women with hypertensive pregnancies. Thirty-seven pregnancies presented HELLP syndrome and required intensive care management (15 had severe preeclampsia, four had chronic hypertension and 18 had eclampsia). The intensive care unit was under the supervision of the anesthesia team and contained seven beds. Therefore, not all the women with HELLP syndrome were transferred to the ICU, because of the lack of intensive care beds. The reasons for admission to the ICU were: worsening of preexisting medical complications, worsening of pregnancy-related diseases and, especially, the need for mechanical ventilation if hypoxemia persisted after oxygen administration (pCO_2_ > 55 mmHg with pH < 725 and pO_2_ < 60 mmHg; altered mental status with impaired airway protection; and respiratory distress with hemodynamic instability).

The maternal clinical characteristics are showed in Table 1. The HELLP syndrome was prepartum in all cases. The mean length of stay in the ICU was 12.83 ± 7.75 days. The neurological examination showed GCS of 11 ± 3.96.

**Table 1:** Clinical characteristics of 37 women with HELLP (hemolysis, elevated liver enzymes and low platelet count) syndrome on admission to an ICU in a tertiary care hospital in Turkey (n = 37)

	Mean (SD)			Range		
Maternal age (years)	29	±	6.98	19	–	42
Gestational age (weeks)	32	±	4.09	25	–	38
Parity	2	±	1.80	0	–	5
Glasgow coma score	11	±	3.96	3	–	15
Systolic pressure (mmHg)	190	±	34.32	140	–	230
Diastolic pressure (mmHg)	114	±	8.94	100	–	130
Platelets (x 10^3^/l)	62,676	±	38,333.37	20,000	–	132,000
Total protein (g/dl)	6	±	0.98	4	–	7
Albumin (g/dl)	3	±	0.56	2	–	4
Blood urea nitrogen (mg/dl)	32	±	11.16	12	–	49
Creatinine (mg/dl)	1.21	±	0.49	0.80	–	3.50
Alanine aminotransferase (U/I)	766	±	644.65	161	–	2,105
Aspartate aminotransferase (U/I)	583	±	667.80	84	–	2,339
Total bilirubin (mg/dl)	3.44	±	2.36	0.60	–	10.50
Lactic dehydrogenase (U/I)	1,773	±	1,226.95	559	–	7,935
Proteinuria	2.56	±	1.09	1	–	4
	**n (%)**					
Epigastric pain	23 (62%)					
Nausea and/or vomiting	31 (84%)					
Headache	6 (16%)					
Visual symptoms	5 (14%)					
Hematuria	8 (22%)					
Cesarean delivery	27 (73%)					
Eclampsia	22 (59%)					
Abruptio placenta	4 (11%)					
Cerebral ischemia-edema	3 (8%)					
Cerebral haemorrhage	15 (40%)					
Acute lung edema	1 (3%)					
Pleural effusion	1 (3%)					
ARDS	2 (5%)					
Severe ascite > 1,000 ml	4 (11%)					
Acute renal failure	4 (11%)					
DIC	2 (5%)					

*HELLP = hemolysis, elevated liver enzymes and low platelet count; ICU: intensive care unit; SD = standard deviation; ARDS: acute respiratory disease syndrome; DIC: disseminated intravascular coagulation.*

One woman with in utero fetal death remained undelivered at the time of her death (case 2, Table 2). Therefore, delivery data were available for 36 patients. Nine of these patients (25%) underwent vaginal deliveries, and 27 patients (75%) had cesarean deliveries. The indications for all the cesarean sections included fetal distress, previous cesarean section, malpresentations, failed induction of labor, unfavorable cervical condition, and deteriorated maternal condition. Twenty-five patients (67%) had spinal anesthesia. The range of platelet counts in this group of patients was 20,000 – 132,000/µl. Among the patients who received spinal anesthesia, eight received platelet transfusions because their platelet count was < 50,000/µl. No patient with vaginal delivery received regional labor analgesia.

**Table 2:** Characteristics of maternal deaths (11) among 37 women with severe hypertensive disorders, all of them with antepartum eclampsia, requiring intensive care management in a tertiary hospital in Turkey

Patient No.	Age (years)	Parity	Gestational age (weeks)	Symptoms presented	Neonatal death	Maternal complications	Delivery during ventilation	Days of mechanic ventilation	Delivery route	Days in ICU
1	23	0	27	Nausea-vomiting, epigastric pain	Yes	Cerebral hemorrhage	Yes	5	CS	5
2	34	0	28	Nausea-vomiting, headache, scotoma, epigastric pain	Yes	Cerebral hemorrhage	No	–	–	–
3	36	2	32	Nausea-vomiting, headache, scotoma	Yes	ARDS	No	4	CS	4
4	37	2	32	Nausea-vomiting, headache, scotoma	Yes	ARDS	No	28	CS	28
5	35	5	30	Nausea-vomiting, epigastric pain	No	Cerebral hemorrhage	No	12	CS	12
6	19	0	38	Nausea-vomiting, epigastric pain	No	Cerebral hemorrhage	No	14	CS	14
7	19	0	34	Nausea-vomiting, epigastric pain	No	Cerebral ischemiaedema	No	15	CS	15
8	20	0	35	Epigastric pain, scotoma, hematuria	No	Cerebral ischemiaedema	No	13	CS	13
9	21	0	35	Nausea-vomiting, epigastric pain, hematuria	No	Cerebral ischemiaedema	No	14	CS	14
10	35	5	30	Nausea-vomiting, epigastric pain, hematuria	No	DIC	No	2	CS	2
11	36	5	30	Epigastric pain, scotoma, hematuria	No	DIC	No	21	CS	21

*ARDS = acute respiratory disease syndrome; DIC = disseminated intravascular coagulation; CS = cesarean section; ICU = intensive care unit.*

A total of 11 cases were supported with mechanical ventilation after the tracheal intubation. The mean duration of mechanical ventilation was 15 ± 5.36 days (range 11-28 days). The mean postpartum stay in the ICU was 13 ± 7.75 days (range 2-36 days). Cerebral hemorrhage was the most frequent maternal complication (40%, 15/37). All of the 37 patients required blood or blood products to correct hypovolemia, anemia or coagulopathy. After diagnosis of acute tubular necrosis (persistent oliguria and rising creatinine levels), standard management of fluid restriction and close monitoring of blood chemistry were instituted. Four women required dialysis: in all cases the indication was uremia. In all of the cases referred for dialysis, there was subsequent improvement in renal function. None of these women required long term-dialysis: the longest treatment lasted four weeks.

There were 11 (30%) maternal deaths. All of these women had been transferred from another institution (Table 2). All of them presented prepartum eclamptic seizure with GCS of 7 ± 4.15. Eight patients were intubated on arrival at our hospital because of respiratory arrest due to eclamptic seizure. Apart from one patient who had intrauterine fetal exitus (case 2), ten cases underwent cesarean delivery of seven live infants. Four patients died because of cerebral hemorrhage. Three patients died because of anoxic encephalopathy and brain death associated with severe cerebral edema, two patients died because of disseminated intravascular coagulation (DIC), and two patients died because of acute respiratory distress syndrome (ARDS). As a result, the most common primary cause of maternal death for this patient population was intracranial hemorrhage (36% of cases). The remaining maternal deaths were due to hypoxic ischemic encephalopathy (27%), DIC (18%) and ARDS (18%) (Table 2). The other patients recovered completely.

## DISCUSSION

It has been proven in many studies that maternal morbidity and mortality have been decreasing since the beginning of last century. Data from developed countries show maternal mortality of about 10% due to preeclampsia and 3–5% due to the HELLP syndrome.^6-8^

However, in Turkey, the maternal mortality rate was 54 per 100,000 in 1999, and most of these deaths were due to severe hypertensive disorders.^9^ This relatively high rate of maternal mortality may be explained by the lack of a standardized antenatal care system in our country. Although obstetricians and public health departments are trying to provide guidelines for the organization of antenatal clinics, so as to standardize antenatal care throughout the country, there is great variation in terms of the frequency and the content of antenatal care visits and in how antenatal care is conducted in Turkey. In addition, the socioeconomic status of our patients, in the northeastern region of the country, is relatively low. Thus, the patients’ financial resources constitute a major limiting factor for the care provider.

The lack of social security for the majority of patients in Turkey results in increased levels of private obstetric practice and hospital provision, at the expense of quality. Substandard care is considered to consist of diagnostic errors or delays, failure to appreciate the severity of the condition and failure to refer patients to senior staff, other disciplines or regional centers with special expertise.^7^

In addition, while acute obstetric morbidity may be sudden and serious, the HELLP syndrome is still sometimes missed or is recognized too late. A delay in making the correct diagnosis involves the risk of life-threatening complications developing or not being recognized in the patient. On the other hand, obstetrics patients have a short period of contact with the ICU. This is supported by objective "severity of illness" measurements such as the APACHE score (Acute Physiology And Chronic Health Evaluation), which demonstrates that obstetrics patients admitted to an ICU score much lower than do other patients transferred to an ICU.^10^ Finally, all of the women in the present study were transferred from another institution and, more importantly, the majority from other cities. The deaths might have been prevented if admission to our hospital had been earlier. Therefore, early diagnosis and optimal emergency management of seizures, hypertension, fluid balance and subsequent safe transfer is essential for minimizing morbidity and mortality.

Cesarean delivery was performed in 75% (27/36) of our cases. Unlike our findings, it has been reported elsewhere that the incidence of cesarean section did not increase with the severity of HELLP syndrome.^11^ Nevertheless, cesarean delivery was most certainly a contributory factor to the rate of successful perinatal outcomes. On the other hand, immediate delivery by the cesarean route did not prevent maternal death for some patients in the present study. Therefore, we suggest that the current therapeutic principle for HELLP syndrome should involve assessment and stabilization, and delivery should be performed soon thereafter.

Patients with HELLP syndrome are at increased risk of DIC, abruptio placentae, pulmonary edema, ARDS, ruptured liver hematoma, acute renal failure and multiorgan failure.^12,13^ Laboratory evidence of DIC was found in only two (5%) out of 37 patients with HELLP syndrome. This result was in contrast to findings by Sibai et al.,^5^ who reported a high incidence of DIC (15-38%) in patients with HELLP syndrome. The most severe forms of renal failure have occurred in cases of complicated preeclampsia, and invariably in cases of HELLP syndrome and abruptio placentae.^14^

It is uncertain whether favorable outcomes reflect aggressive fluid and management protocols or the natural history of the condition. The risk of requiring dialysis was highest among our patients for whom the creatinine level doubled in the first 24-48 hours following hospital admission, in the presence of a major complication of preeclampsia.

According to a study by Isler et al.,^15^ the most common primary cause of maternal death was cerebral hemorrhage (36% of the cases). In a study on all the obstetrics patients treated in ICUs in three French regions over a one-year period, it was shown that hypertensive disease was the most frequent diagnosis (26%) and hemorrhage accounted for 20% of ICU admissions.^16^ Another study in a large liver unit in the United Kingdom showed that, over a ten-year period, 46 women developed hepatic dysfunction that was severe enough to require admission to the liver failure unit, while 15% had HELLP syndrome. Infectious complications occurred in 29% of the patients with HELLP, but there were no maternal deaths associated with HELLP.^17^

Maternal mortality is low in developed countries, and therefore the development of a severe morbidity-mortality ratio might be more useful for evaluating the effect of new treatments or management guidelines. Risk adjustment would be required to account for differences in risk profile between maternity units.^18^ However, the maternal mortality rate in developed countries is not an accurate reflection of the quality of care for most pregnant women, because of the rarity of maternal death.^19^ It has been suggested that near-miss maternal mortality, defined as women requiring transfer to an ICU, is a more comprehensive supplement to the information provided by reviewing maternal mortality^20^ In our unit, hypertension was the main indication for admission to the ICU.

## CONCLUSIONS

Because of the high maternal mortality and morbidity found in association with the HELLP syndrome, standard antenatal follow-up protocols should be applied, so as to obtain early diagnosis. There need to be improvements in the arrangements for transfer without delay to obstetric departments where the staff has expertise in this field.
